# Failure to Use and Sustain Male Condom Usage: Lessons Learned from a Prospective Study among Men Attending STI Clinic in Pune, India

**DOI:** 10.1371/journal.pone.0135071

**Published:** 2015-08-13

**Authors:** Seema Sahay, Swapna Deshpande, Shilpa Bembalkar, Mahesh Kharat, Aparna Parkhe, Radhika G. Brahme, Ramesh Paranjape, Robert C. Bollinger, Sanjay M. Mehendale

**Affiliations:** 1 National AIDS Research Institute, Pune, India; 2 CYTEL, Pune, India; 3 Johns Hopkins University, Baltimore, Maryland, United States of America; 4 National Institute of Epidemiology, Chennai, India; University of Kwazulu-Natal, SOUTH AFRICA

## Abstract

**Background:**

Sustained or consistent use of condoms by men remains a challenge. A study was carried out to identify factors associated with failure to use condoms consistently by men attending STD clinics in Pune, India.

**Method:**

Among 14137 STI clinic attendees, 8360 HIV sero-negative men were enrolled in a cohort study. The changes in condom usage behavior were studied among 1284 men who returned for first scheduled quarterly follow up, 309 reported consistent condom use at the time of enrollment in the cohort. Data pertaining to heterosexual men practicing high risk behavior were analyzed to identify factors associated with change in condom use behavior using logistic regression model. Demographic, behavioral and biological factors observed to be associated with condom use were fitted in five Cox proportional hazards models to calculate hazard ratios and their 95% confidence intervals to identify independent predictors of failure to sustain condom use behavior.

**Results:**

The univariate analysis showed that men who were 30 years or older in age (p = 0.002) and those who did not have contact female sex worker (FSW) were more likely to fail to sustain consistent condom use. However both these factors did not show significant association in multivariable analysis. Marital status and contact with Hijra (eunuch) in lifetime were associated with failure to change in their condom use behavior [AOR 0.33 (CI 0.13–0.82; p = 0.017)]. During the follow up of 2 years, 61 events (15.5 per 100 person years, 95% CI 12.3–19.5 years) of ‘failure of condom use’ were recorded despite counseling. Older age, contact with non CSW partner and presence of genital ulcer disease / discharge syndrome were significant predictors of failure to sustain condom use.

**Discussion:**

Married monogamous older men, who report contact with sex worker and present with genital ulcer disease are at risk of failure to use condom after first exposure to voluntary HIV counseling and testing. This is a scenario of primary prevention program. Condom promotion and counseling needs to be reinforced through follow up counseling among this population.

## Introduction

In developed countries [1,2] and India, the bacterial sexually transmitted infections (STI) like chancroids and gonorrhea are declining, while viral STI like HPV and herpes genitalis are commonly prevalent. According to National AIDS Control Program of India, approximately 30% of core group populations practicing high risk behavior such as female sex workers (FSW), men having sex with men (MSM) and injecting drug users (IDU) suffer from some reproductive tract infections (RTI) or STI in a calendar year [3]. Men having multiple sex partners and those presenting with sexually transmitted infections (STI) act as an important bridge population for spread of HIV from core groups to the general population [4]. Decline in HIV among sex workers has been attributed to sustained targeted interventions [5, 6,7]. India has the third largest number of people living with HIV in the world: 2.1 million at the end of 2013 with an adult prevalence of 0.3%-which accounts for about 4 out of 10 people living with HIV in the region [8, 9]. Of all HIV infections, 39% (930,000) are among women. In India, sexual transmission is responsible for 87.4 percent of reported HIV cases [10].

Although condoms are known to be effective in preventing sexually transmitted infections (STIs) including HIV; consistent condom use has remained a challenge. In 1987, almost one hundred years after the invention of the male rubber condoms, the US Surgeon General recommended their use for HIV prevention and they have proved to be efficacious in preventing HIV transmission [11]. However, their effectiveness at the population level is affected by less acceptability and inconsistent usage which can be partner-dependent [12, 13]. A recent study among 16–25 years old Australian youth reported the predictors of STI history in participants who had sexual intercourse ever, was associated with ‘never’ or sometimes’ condom use [95% CI AOR 1.48 (1.02, 2.16) P 0.04] [14]. A systematic review of 34 studies showed condom non-use to be one of the common barriers to HIV testing in both low and high income countries [15]. Another review highlights the need for behavioral risk reduction and adherence as essential components of any biomedical approach including condom use [16]. India’s National AIDS Control Program emphasizes on promoting condom use during commercial sex [4]. However, despite vulnerability and advocacy, condom use in the marital setting has remained sub-optimal [17]. Non-use of condom among women in India has been linked to socio-cultural context where emphasis needs to be more on family health rather than contraception [18]. A survey among 2408 young men revealed low condom use among men (27%) and women (7%) during premarital sex settings in India [19].

1. Evidence suggests that use of condoms by men during every act of sexual intercourse is protective against HIV transmission and they need to develop condom use habit at the first sexual intercourse itself [20,21]. Since the year 2000, several studies have shown that condoms are effective in preventing STIs among men and women[22]. Following introduction of 100% condom use programme in sex work settings in Thailand, a 95% drop in common curable STIs during the 1990s was reported [23]. In an African study, consistent condom use showed significant reduction in HIV, syphilis and gonorrhea/ chlamydial infections [24]. There is paucity of literature regarding factors affecting the sustainability of consistent condom usage. Changing human behavior is a challenge especially when behavior modifications are required for disease prevention [25]. Condom use by people and the strategies to promote condom use have not changed in the last few decades [26,27]. Recent evidence of non-adherence to various prevention options reported in clinical trials conducted worldwide has led to our interest in analyzing STI cohort data set to learn about non adherence to condom usage as a prevention option. Our study remains pertinent even today as condom use continues to be a challenge [28] and needs to be appropriately positioned even with emergence of newer technologies like vaccine, vaginal microbicides and prophylaxis. It could be a function of two primary components: a) Accepting the advice regarding condom use and actually use them [positive behavior change] and b) Continuing to use condoms at all times even with STIs [sustaining positive behavioral change]. We investigated these two dimensions of condom use among men attending STI clinics in Pune, India. We feel that findings of this study may guide in formulating appropriate guidelines and strategies to increase condom use as well as ensure its consistent use for prevention and control of STI and HIV in India. In a cohort of men attending STI clinics in Pune, India, we assessed the change and sustenance of condom use behavior and studied the predictors of failure to sustain consistent condom use during peno-vaginal sex.

## Methods

### Ethics

The study and informed consent form was approved by the Ethics Committee of the National AIDS Research Institute and Institutional Review Board of Johns Hopkins University Joint Committee on Clinical Investigation. Written informed consent was obtained from all study participants. The consent was in local language and it was administered by trained counselors. For the participants who were illiterate, the consent was read out and was explained in the presence of an impartial witness. The consent was either signed by the participant or thumb impression was obtained in case of illiterate participant in the presence of witness. The copy of informed consent was offered to all participants. The informed consents were kept in a separate file under lock and key. Participants who refused to take the copy of informed consent form; their consent form was also filed separately and stored under lock and key.

### Study Design

Prospective, clinic-based, cohort study.

### Study Population and Period

Three STI clinics (1 municipal STI clinic, 1 STI clinic in a state government teaching hospital, and 1 health care center in a red light area) are operated in Pune, Maharashtra by the National AIDS Research Institute (NARI). Between May 1998 and August 2002, men and women attending any of the above-mentioned STI clinics were screened for HIV-1, HIV-2, and STI diagnosis. This study pertains to men attending STI clinics.

### Study Procedure and Instruments

Individuals aged 18 years or older, and willing to provide informed consents were enrolled. According to the lead counselors of the study, there were no refusals for participation. A structured interviewer-administered questionnaire was used to obtain data on demographics, medical history, sexual and other risk behaviors, knowledge of HIV and condom use practices at baseline and at each quarterly follow up visit for a period of two years. Participants underwent physical examination and were tested for HIV and STI [29]. All participants received HIV pre and post-test counseling by trained and qualified counselors. Risk reduction counseling and condom demonstration were done. The participants were counseled about importance of receiving test reports and follow up. There was no consent for home visit; hence home visits for retention were not done. Clinical and laboratory diagnoses of STIs were made following the national guidelines and standard treatment was provided [30]. For the purpose of this analysis, HIV sero-negative men who reported having heterosexual partners at enrollment at the STD clinic were considered.

### Operational definitions used in the study

Condom use with regular or non-regular partners has been used as indicator in several surveys [31]. In this study, reported condom use during sex with regular or irregular partner was studied. The question asked was ‘Since your last visit did you have any sexual partners who were female; eunuch or male- non eunuch? Type of sex (vaginal, oral, and anal) with each partner, frequency of condom use (never, sometimes, and always) and type of partner (commercial sex worker, non-commercial regular or casual sex partner or HIV + partner) was explored.

#### Defining the study variables pertaining to condom use

**Fig 1:**
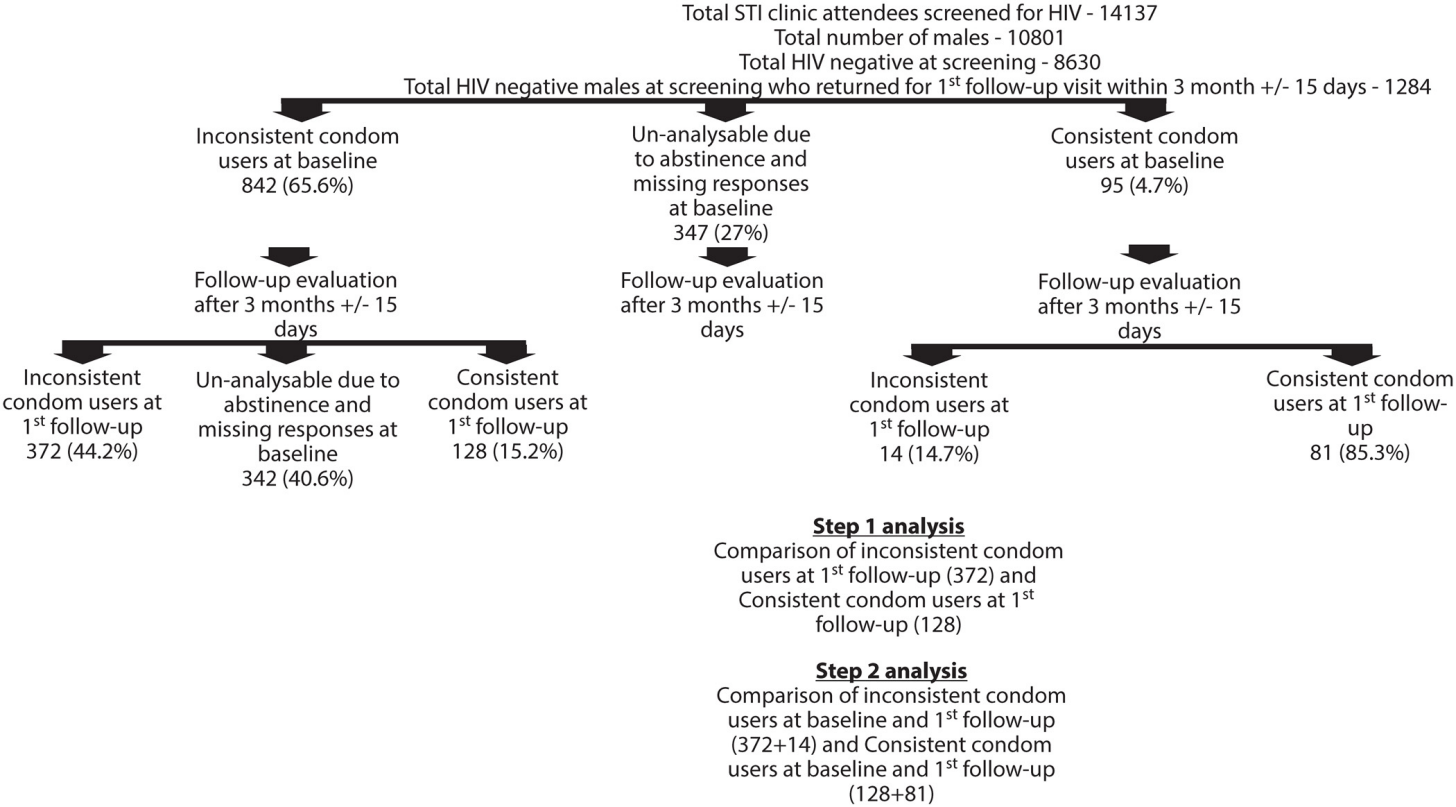
Showing the study participant characteristics in step 1 and step 2 analysis

Three groups of men came at baseline as shown in the schema (Fig 1):

1. Men who reported inconsistent condom use
2. Men who reported consistent (always) condom use
3. Men who reported to be abstinent or non-responders [no response]

At enrollment, i.e. at first follow up after 3 months, all the men who reported always / consistent condom use were considered as consistent condom users and analysis was done to understand the sustainability of condom use behavior among these men. Therefore at first follow up, we had men who were inconsistent condom users, abstinent/ non responders and men who were consistent condom users. The operational definitions for condom use are as follows:

#### Consistent condom use

Using condoms ‘always’ within a period of 3 months ±15 days from the enrollment visit or continuing to ‘always’ use condoms until the first follow-up visit among those who reported using condoms ‘always’ at baseline. The question was asked about frequency of condom use (never, sometimes, always) with sex partner with in the last 3 months.

#### Inconsistent condom use

Using condoms ‘never or sometimes’ within a period of 3 months ±15 days from the enrollment visit or using condoms ‘never or sometimes’ until the first follow-up visit.

#### Positive condom use behavior

Change from inconsistent condom use (never/ sometimes) to consistent condom use.

#### Sustained condom use

‘Consistent’ condom use with any partner/ s from baseline to the entire study period of 2 years among those who had positive condom use behavior at the first 3 monthly follow-up visits.

#### Failure of condom use

Change of condom use behavior from ‘consistent condom use’ to ‘inconsistent condom use’.

### Study population

All STI patients enrolled in the study who returned for 1^st^ follow-up visit were included in the analysis to get clue on the outcome primary prevention which was HIV counseling and testing and treatment of current STI. Of the total of 14137 men and women attending STI clinics during the study period, 10801 (76.4%) were men. All were screened for HIV. Of the total men, 8630 were HIV sero-negative and 1284 of them returned for their first three monthly follow-up visit. These 1284 men were considered for the present study. All of them received primary prevention counseling and HIV testing at baseline and they were called for the next follow up scheduled after 3 months±15 days.

Statistical Analysis: Analysis was performed in two steps as summarized in Fig 1: Showing the study participant characteristics in step 1 and step 2 analysis.

We performed two different types of analyses as follows:

**Step 1 analysis.** For step 1 analysis, comparison of characteristics of inconsistent condom users (372/842) and consistent condom users at 1st follow-up (128/842) was made. Data on rest of the individuals’ was not included because of non-response, missing data or reported abstinence. Demographic, behavioral, biological and clinical variables were used in the univariate and multivariable logistic models to identify predictors of failing to change to positive condom behavior. Rationale of covariates selected was based on researcher’s interest and opinions of expert clinicians in the field, epidemiologist and social scientist. The enter method by self-selection was used for univariate and multivariable logistic regression. Cox and Snell R square method was used to check model adequacy.
**Step 2 analysis.** In addition to 128 patients who changed to positive condom behavior in step A; men (who already had positive condom use behavior at baseline (were using condoms ‘always’) and continued to sustain positive condom behavior until the first follow-up 81/95) were considered for step 2 analysis. We analyzed data on condom use and associated factors among these 209 to identify ‘those who sustained consistent condom use’ and 386 who ‘failed to sustain condom use’ for the total duration of follow up of 2 years (Fig 1). We employed Cox proportional hazards models with time dependent covariates [32] to calculate hazard ratios with their 95% confidence intervals to identify predictors of failure to sustain consistent condom use. The proportional hazards assumption was checked using Schoenfeld residuals [33].

We hypothesized that demographic factors, sexual behavior and biological factors could affect condom use differentially and hence we used multiple models to verify the predictors of lack of condom use in our study population. In all, 7 models were constructed using baseline values of various covariates [29, 30, 34, 35, 36] as follows:

Model 1—Four demographic variables, viz. age, education, employment, religion
Model 2—Variables used in Model 1 and marital status
Model 3—Variables used in Model 2 and history of alcohol consumption
Model 4—Variables used in Model 2 and 3
Model 5—Variables used in Model 4 and two variables reflecting sexual behavior that might influence condom use [contact with non-CSW partners in last 3 months and contact with Hijras or CSW partners in last 3 months]
Model 6—Variables used in Model 4 and two variables reflecting biological factors that might influence condom use [Presence of Genital Ulcer Diseases (GUDs), Genital Discharge Diseases (GDs) or GUDs / GDs in last 3 months]
Model 7—Variables used in Model 5 and 6.

Two sided probability values <0.05 were considered statistically significant. All statistical analyses were conducted using STATA 10.0 (StataCorp, College Station, Texas) and SPSS 15.0 statistical software (SPSS Inc., USA)

## Results

In step 1 analysis, comparison of 128 men who adopted positive condom use behavior and 372 who did not was made. “Table 1” shows that in univariate analysis, men who were 30 years or older and who were monogamously married were more likely to fail to change to positive condom behavior [OR: 1.94 (CI: 1.29–2.91; p = 0.002)] and [OR: 4.02 (CI: 2.60–6.21; p<0.001)] respectively.

**Table 1:** Factors associated with failure to change from inconsistent condom use to ‘positive’ condom use behavior among male STD patients.

Characteristic	Failed to convert to consistent condom use	Converted to consistent condom use	Univariate analysis		Multivariable Analysis	
	n = 372	n = 128	Odds Ratio (95% CI)	P value	Odds Ratio (95% CI)	P value
**Age**						
< 30 Yrs	160 (43.0)	76 (59.4)	1 (Ref)			
≥ 30 Yrs	212 (57.0)	52(40.6)	1.94 (1.29, 2.91)	0.002**	1.47 (0.68, 3.18)	0.33
**Religion**						
Hindu	314 (84.4)	107 (83.6)	1 (Ref)		1 (Ref)	
Buddhist	32 (8.6)	14 (10.9)	0.78 (0.40, 1.52)	0.462	0.79 (0.30, 2.06)	0.632
Other	26 (7.0)	7 (5.5)	1.27(0.53, 3.00)	0.593	1.78 (0.50, 6.33)	0.371
**Marital status**						
Others‡	67 (18.0)	60 (46.9)	1 (Ref)		1 (Ref)	
Monogamously married	305 (82.0)	68 (53.1)	4.02 (2.60, 6.21)	< 0.001**	3.93 (1.59, 9.77)	0.003**
**Living Away from Home**						
No	319 (87.6)	95 (74.8)	1 (Ref)		1 (Ref)	
Yes	45 (12.4)	32 (25.2)	0.42 (0.25, 0.70)	0.001**	1.15 (0.48, 2.72)	0.759
**Education**						
Illiterate	44 (11.8)	14 (10.9)	1 (Ref)		1 (Ref)	
Primary schooling	177 (47.6)	62 (48.4)	0.91 (0.47, 1.77)	0.778	0.93 (0.34, 2.50)	0.879
Secondary & high school	115 (30.9)	37 (28.9)	0.99 (0.49, 2.00)	0.975	0.56 (0.20, 1.62)	0.285
Vocational course	36 (9.7)	15 (11.7)	0.76 (0.33, 1.79)	0.535	0.64 (0.18, 2.25)	0.486
**Employment Status**						
Unemployed	27 (7.3)	13 (10.2)	1 (Ref)		1 (Ref)	
Employed	345 (92.7)	115 (89.8)	1.44 (0.72, 2.89)	0.299	0.35 (0.11, 1.06)	0.064
**No of female partners in lifetime**						
One	34 (9.7)	9 (7.6)	1 (Ref)		1 (Ref)	
Two or more	318 (90.3)	110 (92.4)	0.76 (0.36, 1.65)	0.494	0.38 (0.13, 1.09)	0.072
**Hijra** ^**1**^ **contacts in lifetime**						
No	333 (89.5)	110 (85.9)	1 (Ref)		1 (Ref)	
Yes	39 (10.5)	18 (14.1)	0.72 (0.39, 1.30)	0.273	0.33 (0.13, 0.82)	0.017*
**Age at 1** ^**st**^ **sex**						
Up to 19 Yrs	224 (60.4)	81 (63.8)	1 (Ref)		1 (Ref)	
Above 19 Yrs	147 (39.6)	46 (36.2)	1.16 (0.76, 1.75)	0.497	0.78 (0.38, 1.59)	0.496
**Diagnosis of HSV**						
No	327 (88.4)	113 (89.7)	1 (Ref)		1 (Ref)	
Yes	43 (11.6)	13 (10.3)	1.14 (0.59, 2.20)	0.69	0.78 (0.26, 2.35)	0.655
**Recent FSW partners**						
No	202 (54.3)	52 (40.6)	1 (Ref)		1 (Ref)	
Yes	170 (45.7)	76 (59.4)	0.58 (0.38, 0.87)	0.008**	0.59 (0.29, 1.23)	0.158
**Reason for clinic visit–Symptoms**						
No	69 (20.4)	24 (22.0)	1 (Ref)		1 (Ref)	
Yes	269 (79.6)	85 (78.0)	1.10 (0.65, 1.86)	0.72	0.98 (0.45, 2.12)	0.948
**Symptoms of genital discharge**						
Absent	284 (76.5)	107 (83.6)	1 (Ref)		1 (Ref)	
Present	87 (23.5)	21 (16.4)	1.56 (0.92, 2.64)	0.097	1.65 (0.73, 3.75)	0.229
**Symptoms of genital warts**						
Absent	213 (96.8)	92 (93.9)	1 (Ref)		1 (Ref)	
Present	7 (3.2)	6 (6.1)	0.50 (0.17, 1.54)	0.229	0.63 (0.14, 2.90)	0.557
**Symptoms of genital ulcer**						
Absent	221 (59.6)	85 (66.4)	1 (Ref)		1 (Ref)	
Present	150 (40.4)	43 (33.6)	1.34 (0.88, 2.05)	0.172	0.66 (0.34, 1.28)	0.215
**GUD**						
No	270 (72.6)	97 (75.8)	1 (Ref)		1 (Ref)	
Yes	102 (27.4)	31 (24.2)	1.18 (0.74, 1.88)	0.48	1.59 (0.72, 3.52)	0.253
**GD**						
No	326 (87.6)	116 (90.6)	1 (Ref)		1 (Ref)	
Yes	46 (12.4)	12 (9.4)	1.36 (0.70, 2.67)	0.364	2.00 (0.64, 6.26)	0.232

^‡^Others include unmarried, divorced, widower and separated.

GUD: Genital ulcer disease, GD: Genital discharge

* Significant at p value < 0.05,

** Significant at p value < 0.01

^1^ Hijra is local term for éunuch

Men who lived away from family [OR 0.42 (CI 0.25–0.70; p = 0.001)] and men who had recent FSW partner [OR 0.58 (CI 0.38–0.87; p = 0.008)] were less likely to fail to use condom consistently. In multivariable analysis, an independent association was observed between married monogamous men and likelihood of failure to achieve positive change in their condom use behavior [AOR 3.93 (CI 1.59–9.77; p = 0.003)]. Contact with Hijra (eunuch) in lifetime was also observed to be independently associated with failure to change their condom use behavior [AOR 0.33 (CI 0.13–0.82; p = 0.017)], the number of respondents having such a behavior was very small.

In step 2 analysis follow-up data on 209 men who reported positive condom use behavior at first follow up visit was analyzed to determine the predictors of sustained consistent condom use behavior. Sixty-one events of ‘failure of condom use’ during the study period of two years were recorded. The incident rate for failure of consistent condom use was 15.5 per 100 person years (95% CI 12.3–19.5 years). “Table 2” summarizes hazard ratios and their 95% confidence intervals as well as p values for failure to sustain positive condom behavior in case of various models studied.

**Table 2:** Cox professional hazards analysis showing risk factors and hazards of inconsistent condom use during follow-up of two years among male patients attending STD clinics.

Model	Covariate major headings	Covariates	Covariate categories	Hazards Ratio, HR, (95% CI)	p value
**Model 1**	**Basic demographic variables**	Age (years)	<30	1	
			≥30	**1.91 (1.09, 3.34)**	**0.024** *
		Education	Primary school	1	
			Secondary and High school	**0.55 (0.31, 0.97)**	**0.039** *
			Vocational training or College	0.79 (0.37, 1.72)	0.558
		Employment	Unemployed	1	
			Employed	1.31 (0.51, 3.38)	0.573
		Religion	Hindu	1	
			‡Others	0.89 (0.47, 1.69)	0.716
**Model 2**	**Basic demographic variables and marital status**	Age (years)	<30	1	
			≥30	**2.22 (1.19, 4.13)**	**0.012** *
		Education	Primary school	1	
			Secondary and High school	**0.50 (0.27, 0.94)**	**0.031** *
			Vocational training or College	0.73 (0.33, 1.65)	0.455
		Employment	Unemployed	1	
			Employed	1.31 (0.44, 3.83)	0.627
		Religion	Hindu	1	
			‡Others	0.91 (0.43, 1.91)	0.801
		Marital Status	Unmarried and $ $ others	1	
			Monogamously married	1.68 (0.96, 2.92)	0.068
**Model 3**	**Basic demographic variables and alcohol**	Age (years)	<30	1	
			≥30	**1.86 (1.06, 3.27)**	**0.030** *
		Education	Primary school	1	
			Secondary and High school	**0.53 (0.30, 0.94)**	**0.030** *
			Vocational training or College	0.76 (0.35, 1.66)	0.49
		Employment	Unemployed	1	
			Employed	1.34 (0.52, 3.46)	0.543
		Religion	Hindu	1	
			‡Others	0.89 (0.47, 1.69)	0.714
		Alcohol	No	1	
			Yes	0.67 (0.20, 2.24)	0.519
**Model 4**	**Basic demographic variables and alcohol and marital status**	Age (years)	<30	1	
			≥30	**2.30 (1.22, 4.32)**	**0.010** **
		Education	Primary school	1	
			Secondary and High school	**0.52 (0.28, 1.00)**	**0.049** *
			Vocational training or College	0.76 (0.34, 1.74)	0.523
		Employment	Unemployed	1	
			Employed	1.29 (0.44, 3.79)	0.644
		Religion	Hindu	1	
			‡Others	0.94(0.44, 1.98)	0.872
		Alcohol	No	1	
			Yes	1.78 (0.36, 8.92)	0.48
		Marital Status	Unmarried and $ $ others	1	
			Monogamously married	1.61 (0.92, 2.85)	0.097
**Model 5**	**Basic demographic variables and alcohol and marital status and sexual behavior**	Age (years)	<30	1	
			≥30	1.66 (0.86, 3.20)	0.133
		Education	Primary school	1	
			Secondary and High school	0.65 (0.33, 1.27)	0.21
			Vocational training or College	1.06 (0.43, 2.63)	0.894
		Employment	Unemployed	1	
			Employed	0.93 (0.30, 2.94)	0.906
		Religion	Hindu	1	
			‡Others	0.54 (0.24, 1.21)	0.134
		Alcohol	No	1	
			Yes	0.59 (0.11, 3.34)	0.554
		Marital Status	Unmarried and $ $ others	1	
			Monogamously married	1.55(0.88, 2.73)	0.132
		Contact with Non-CSW partners in last 3 months	No	1	
			Yes	**9.09 (4.45,18.57)**	**<0.001** **
		Contact with Hijra or CSW in last 3 months	No	1	
			Yes	1.89 (0.94, 3.81)	0.076
**Model 6**	**Demographic variables and alcohol and marital Status and biological variables**	Age (years)	<30	1	
			≥30	**2.42 (1.27, 4.62)**	**0.007** **
		Education	Primary school	1	
			Secondary and High school	0.56 (0.29, 1.08)	0.085
			Vocational training or College	0.87 (0.38, 2.03)	0.757
		Employment	Unemployed	1	
			Employed	1.22 (0.41, 3.64)	0.721
		Religion	Hindu	1	
			‡Others	0.94 (0.43, 2.04)	0.88
		Alcohol	No	1	
			Yes	1.46(0.16, 13.29)	0.738
		Marital Status	Unmarried and $ $ others	1	
			Monogamously married	1.63 (0.91, 2.95)	0.102
		Circumcision	No	1	
			Yes	0.84 (0.19, 3.72)	0.817
		Presence of GUDs in last 3 months	No	1	
			Yes	1.77 (0.69, 4.53)	0.234
		Presence of GDs in last 3 months	No	1	
			Yes	0.67 (0.08, 5.93)	0.722
		Presence of GUDs / GDs in last 3 months	No	1	
			Yes	2.00 (0.88, 4.53)	0.098
**Model 7**	**Demographic variables and alcohol and marital Status and sexual behavior and biological variables**	Age (years)	<30	1	
			≥30	1.82 (0.91, 3.61)	0.088
		Education	Primary school	1	
			Secondary and High school	0.62 (0.31, 1.24)	0.176
			Vocational training or College	0.99 (0.39, 2.52)	0.985
		Employment	Unemployed	1	
			Employed	0.86 (0.27, 2.76)	0.795
		Religion	Hindu	1	
			‡Others	0.54 (0.23 1.26)	0.153
		Alcohol	No	1	
			Yes	0.51 (0.04, 6.26)	0.602
		Marital Status	Unmarried and $ $ others	1	
			Monogamously married	1.62 (0.89, 2.96)	0.118
		Contact with Non-CSW partners in last 3 months	No	1	
			Yes	**10.09(4.78, 21.28)**	**<0.001** **
		Contact with Hijra or CSW in last 3 months	No	1	
			Yes	1.64 (0.77, 3.51)	0.201
		Circumcision	No	1	
			Yes	0.96 (0.21, 4.48)	0.962
		Presence of GUDs in last 3 months	No	1	
			Yes	2.47 (0.97, 6.27)	0.057
		Presence of GDs in last 3 months	No	1	
			Yes	0.27 (0.03, 2.53)	0.251
		Presence of GUDs / GDs in last 3 months	No	1	
			Yes	2.31 (1.00, 5.38)	0.051*

^‡^ Buddhist, Christian, Muslims and Others

^$ $^ Widowed, divorced and separated.

NA: Not applicable

* Significant at p value < 0.05,

** Significant at p value < 0.01

Multiple models were used to understand effects of absence/ presence of various risk factors on inconsistent condom use behavior to demonstrate a comparative impact of factors under the respective domains of demographic, behavioral, sexual behavioral and biological covariates respectively. In the first four models, men, who were 30 years or older were more likely to fail to sustain positive condom use behavior. As the covariates were added to models, the hazard ratio of age kept increasing for model no. 1 to model no.4 and model no. 6 showed a steady increase from 1.9 to 2.41 indicating older age as a predictor for inconsistent condom use. Those who were educated at the level of secondary school or high school were more likely to sustain consistent condom use behavior during the follow-up period of 2 years. Interestingly, both these variables lost significant relationship with sustained condom use behavior when additional variables related to sexual behavior were added in models 5 and 7.

STI patients who had primarily non-commercial sex worker contacts during the past 3 months were nearly 9 times likely to fail to sustain positive condom use behavior [Model 5]. Age of more than 30 years was found to be associated with failure to sustain positive condom use behavior [Model 6].

The final model [model 7] inclusive of all the demographic, sexual behavior related and biological variables showed that STI patients who had primarily non-commercial sex worker contacts (probably their regular partners) during the past 3 months or who suffered from genital ulcer diseases or genital disease (GUD/ GD) in the past 3 months were more likely to fail to sustain positive condom use behavior.

## Discussion

This study reports predictors of failure to use condom consistently among men attending STI clinics in Pune, India in a primary prevention setting of voluntary HIV counseling and testing. Rate of return for follow-up for clinical evaluation and consistent condom use were observed to be low in the study population as reported earlier [37] Low retention could be attributed either to patients’ choice, inadequacy in motivational counseling and existing stigma and discrimination during the study period. Owing to extreme stigma existing during that period in India [38], study had limitation of having no provision for home visit. Retention among male patients is reported to be a challenge [39]. Strategies such home visit or telephonic contact for ensuring adequate follow-up need to be stressed. Better follow-up provide more opportunities for counseling interventions which might result in overall improvement and retention in primary prevention programs. Use of multiple models in this study provides an opportunity to target patients with specific demographic and other characteristics to implement appropriate interventions that could improve condom use in similar populations.

Several studies have reported protective role of condom use against HIV acquisition. There is paucity of literature about situations and reasons for failure to use condom consistently. The setting of a prospective cohort study helped us to compute hazards ratios and predictors of sustained positive condom use behavior. Older men failed to sustain consistent use of condom. World over, similar observations regarding risky behavior with increasing age have been reported [40, 41, 42, 43]. This finding has programmatic implications for HIV and STI prevention at Integrated Counseling and Testing Centers (ICTCs) and other clinical set ups where HIV/ STI risk reduction counseling is being routinely undertaken. Additional efforts, such as periodic reinforcement counseling and ensuring sustainability of condom use among older men are recommended. In the 2 years’ follow up data, higher education, presence of GUD and reported contacts with commercial sex workers were the risk factor for inconsistent condom use among the study participants. This revelation highlights the existing condom use programs that are targeted towards key populations whereas sex workers’ client centered approach of condom promotion emerges critical. Sex workers in Africa have reported that men do not want to use condoms [44]. A recent study among gay men, female sex workers and clients have shown that condom use behaviors among high risk groups is contextual decision and it is not a planned behavior [45].

Men having extramarital and multi-partner sex are exposed to risk of acquiring HIV and STI [46]. Men tend to use condoms during commercial sex, but this behavior is inconsistent [5,29]. Condoms are also often regarded as irrelevant in long-term relationships due to lack of perception of risk among monogamous married men who prefer not to use condoms consistently with their regular partners [47]. Heterosexual transmission is the major mode of HIV transmission in India [48] with most married women living with HIV having acquired it from their spouses [49, 50]. Being married and monogamous was another factor of failure to sustain consistent condom use behavior among these men who were attending STI clinics in India. A large study in Britain also reported that 80% of sexually active, never married respondents used condoms ‘*to protect against HIV and other STI’* in contrast to 1.8% of married respondents [51]. The negative condom use behavior among married monogamous men finding reinforces the need for targeting married monogamous men attending STI clinics to inculcate ‘condom use habit’ amongst them which would benefit the spouse as well as the casual partner. Casual and short partnerships have been linked to higher probability of condom use [52] and this study brings out the higher likelihood of inconsistent condom use behavior in long standing relationship. Men living away from family and those who had contacts with Hijras (eunuch) or with female sex workers were less likely to fail to use condom consistently. This finding shows the perception for self-risk but men in this study lacked perception of risk to their married partners. This is a matter of concern today also because the current Indian HIV epidemic has diversity of risks wherein currently the HIV epidemic is driven by MSM [53]. Owing to socio cultural reasons ‘men’ get married despite different sexual orientation [54, 55]. In order to remain hidden in the current social and political setting in India, married MSM often indulge in hurried covert increasing their risk and risk to their regular married partners [56]. The primary reason driving condom use in India is preventing unwanted pregnancy and despite high awareness of STDs/ HIV/ AIDS in India, condom use as a means of protection against STI and HIV/ AIDS is still low [57]. Men should also be made to clearly understand the risk they may pose risk to their marital partners and the need to use condoms during each act of sex whether in personal marital or commercial setting. Prevention program needs to develop newer strategies for condom promotion among men especially targeting men who present themselves with genital diseases and those who are married and monogamous. Focused efforts to create awareness regarding efficacy of consistent condom use in HIV and STI prevention are necessary. Interventions need to focus on combined behavioral and structural approaches and the program needs to sensitize the bridge populations coming in sexual contract with the high risk populations on condom use.

The limitation of our study was our inability to analytically explore the relationship between presence of GUD/ GD and failure to sustain consistent condom use. Despite consistent condom use at baseline, these men were attending STI clinic. This could indicate either condom use failure, social desirability bias in reporting condom use or failure to use condom due to discomfort while using condoms in presence of STI. National data on genital ulcers from the 2007 Zambia Demographic and Health Survey showed that 57% respondents reported sex after onset of symptoms and only 15% reported consistent condom use [58]. Sexual behavior among men presenting with STI including GUD/ GD needs to be explored in India. Another limitation was that this study was conducted among men attending STI clinics; this population may not be comparable to the overall male population of Pune, Maharashtra, or India. The follow up rate of male STI clinic attendees was 15%. This has a limitation of the study. However, those who returned provide us adequate sample size to study the predictors of lack of condom use and more realistically reflect the real life situation.

## Conclusions

Condoms are inexpensive, easy to store, carry and use and do not require medical consultation. The target should be to emphasize correct and consistent usage of condom. Behavior change especially pertaining to consistent condom use has been a challenge and sustaining such a behavior is an additional challenge. Only one time voluntary counseling and testing might fail in case of men who are married monogamous, older men reporting contact with female sex worker/ s, Hijras and/ or presenting with GUD as this may lead to condoms non-use. This calls for continuum of prevention care by instituting follow-ups in the HIV prevention programs. In the STI clinics, reinforcement of educational messages and condom promotion through follow ups among this group of men is essential for sustained behavior change. Programs have focused on condom use among sex workers, it would be more relevant to also review the mediating mechanism of condom use variables among men especially the client of sex workers. These could be the men who do not use condoms with non FSW sex partner owing to false sense of security, but the program focuses mainly on key high risk populations. The message should be to acquire condom use habit irrespective of the partner.
